# Detection of Android Malware in the Internet of Things through the K-Nearest Neighbor Algorithm

**DOI:** 10.3390/s23167256

**Published:** 2023-08-18

**Authors:** Himanshi Babbar, Shalli Rani, Dipak Kumar Sah, Salman A. AlQahtani, Ali Kashif Bashir

**Affiliations:** 1 Chitkara University Institute of Engineering and Technology, Chitkara University, Rajpura 140401, Punjab, India; 2 Department of Computer Engineering and Application, GLA University, Mathura 281406, Uttar Pradesh, India; dipak.sah@gla.ac.in; 3 Department of Computer Engineering, College of Computer and Information Sciences, King Saud University, P.O. Box 51178, Riyadh 11543, Saudi Arabia; salmanq@ksu.edu.sa; 4 Department of Computing and Mathematics, Manchaster Metropolitian University, Manchaster M15 6BH, UK; dr.alikashif.b@ieee.org

**Keywords:** android malware, recommender system, Internet of Things, static analysis, machine-learning algorithms, K-nearest neighbor

## Abstract

Predicting attacks in Android malware devices using machine learning for recommender systems-based IoT can be a challenging task. However, it is possible to use various machine-learning techniques to achieve this goal. An internet-based framework is used to predict and recommend Android malware on IoT devices. As the prevalence of Android devices grows, the malware creates new viruses on a regular basis, posing a threat to the central system’s security and the privacy of the users. The suggested system uses static analysis to predict the malware in Android apps used by consumer devices. The training of the presented system is used to predict and recommend malicious devices to block them from transmitting the data to the cloud server. By taking into account various machine-learning methods, feature selection is performed and the K-Nearest Neighbor (KNN) machine-learning model is proposed. Testing was carried out on more than 10,000 Android applications to check malicious nodes and recommend that the cloud server block them. The developed model contemplated all four machine-learning algorithms in parallel, i.e., naive Bayes, decision tree, support vector machine, and the K-Nearest Neighbor approach and static analysis as a feature subset selection algorithm, and it achieved the highest prediction rate of 93% to predict the malware in real-world applications of consumer devices to minimize the utilization of energy. The experimental results show that KNN achieves 93%, 95%, 90%, and 92% accuracy, precision, recall and f1 measures, respectively.

## 1. Introduction

The IoT is the most significant digital hot trend, bridging the real and digital spheres. Individuals, things, machines, and the internet are becoming increasingly connected, resulting in the formation of novel business models and novel interconnections between humans and the rest of the world. IoT devices have become an attractive target for hackers who take advantage of the verification of vulnerabilities, obsolete firmware, and malware to negotiate IoT devices due to the difficulty in formulation and construction in both software and hardware as well as a lack of security features and skills [1]. As a result, the main target of the hackers is to hack the data of the central server, i.e., the cloud server. Between January 2021 and June 2023, 1.51 billion IoT vulnerabilities were reported, most of which used the Telnet remote access protocol (https://techinformed.com/iot-in-2023-and-beyond/#:~:text=According%20to%20Statista%2C%20there%20are,people%20worldwide%20(eight%20billion, accessed on 19 July 2023). According to Kaspersky, an antivirus and computer security network operator, IoT cyberattacks will more than double year over year in the first half of 2021. With the fast use of IoT technology in the industry, the number of attacks will continue to rise indefinitely. The need for recommendation systems is increasing due to the time shortage and usage of various types of applications [2]. To protect the cloud server from remote devices, a recommender system is required that can predict malware and recommend the server to block those devices from transmitting the data [3]. Malware (malicious software) is among the most significant threats facing IoT devices. The fundamental definition of malware is any harmful application containing malicious files with the wicked aim of gaining unauthorized access and performing no authorized or acceptable activities while breaching the three primary security requirements of secrecy, authenticity, and reliability. The Mirai virus family launched one of the biggest and most influential DDoS attacks in recent years on Dyn (a major US DNS service provider) in November 2017 [4]. Over 1.5 million IoT devices were affected, and the malware targeted numerous famous internet services such as Gmail, Amazon, and others. A cybersecurity developer team in Moscow acquired 121,600 IoT malware samples in 2019, roughly triple the 35,618 samples it acquired in 2018, and over 130,000 variant IoT malware samples whose attacking tactics were smartly evolved were recommended security on these devices [5].

In the proposed approach, supervised learning algorithms such as decision trees, random forests, and neural networks are used to train a model on a dataset of known Android malware attacks [6]. The model can then be used to predict whether an incoming request is an attack or not based on its features and hence recommend the server to block them. Another approach is to use unsupervised learning algorithms such as clustering and anomaly detection to identify patterns and anomalies in network traffic that could indicate a malware attack. These techniques can be especially useful when dealing with unknown or novel attacks [7].

Malware is a software developed by cybercriminals to obtain access to or harm a computer or network even without the victim’s awareness [8]. Malware categories keep evolving, even though there was a 45 percent decline in malware worldwide in 2021. Several malware categories have evolved into hybrids as they employ equivalent malware potential attacks, like using logic explosives, which are pre-programmed attacks that are often provoked by victims themselves, phishing and social engineering tactics to deliver malware directly to victims, or mobile malware, which specifically targets mobile devices [9,10]. In the most recently reported period, 4.8 billion malware attacks were carried out, which was down from 5.6 billion attacks in the previous year. The prediction of Android malware on IoT devices is a case study for the proposed methodology [11]. Android malware is malware that is particular to the Android OS and affects or exploits data from such an Android-based mobile device. Trojans, Spyware, Adware, Worms, Botnets, and Rootkits are some of the types of malware which can be transmitted to the cloud server [12]. The importance of this activity is illustrated by the fact that Android has started growing to be the most popular cellphone operating system in the globe, with over a billion people around the world, whereas the number of malware precisely targeting them has increased at the same time: anti-virus vendors detect thousands of additional malware samples every day with little or no end in sight. We will concentrate our investigation on the DREBIN [13] dataset, which is a machine-learning method that aspires static analysis to predict Android malware directly on the mobile device. This prediction of Android malware has a problem regarding classification, which indicates the class of an application [14]. The different classes comprised two categories: either the class is benign or malware application, which is signified by 0 or 1.

**Figure 1:**
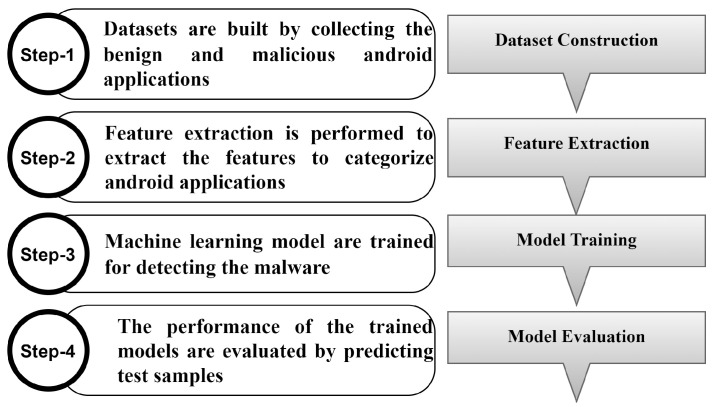
Steps for prediction of Android malware in machine learning in recommender systems.

The essential key aspects of Android are its use as a broadcast receiver and content provider. A broadcast receiver is used for sending the data amongst the various applications. This is used by the malware developers that can track the activities that contain sensitive information [15]. It refines, receives, and responds back to the departing broadcasts which later permits the Android applications to have the capability to respond to external activities like receiving text messages or audio/video calls, while the content provider helps the Android malware exchange the data for seeking permission to execute the malicious behavior [1]. These permissions and the key aspects used in building the Android application are required to be registered in the AndroidManifest.xml file, which is mostly deployed as the auxiliary indicator to be incorporated with other analysis techniques for the prediction of malware. The various steps to detect the Android malware in machine learning are shown in Figure 1.

Therefore, feature extraction enhances the efficiency of the prediction of malware by reducing duplicates and features that are not relevant to the prediction of Android malware. The extraction of features deploys static, dynamic, and hybrid analysis methods for the prediction of Android malware [16,17]. Researchers and engineers have suggested many security techniques to prevent malware attacks from consumer devices and hence to the cloud server, including static analysis, dynamic analysis, and artificial intelligence. Nevertheless, data science has emerged as a potential field in cybersecurity, as numerical simulations based on information enable the discovery of insights that can assist in the prediction of malicious behavior. The static analysis involves examining Android files and accessing information such as the requested permissions, opcode sequences, and API calls, among other things. For several additional features that are straightforward to retrieve, static detection is commonly employed in the realm of malware prediction for Android [18]. Dynamic analysis is the technique in which the program is run in a sandbox environment, and the behavior of the API call sequencing, system calls, traffic patterns, and CPU information is tracked to analyze the flow of data throughout the system’s execution, revealing the true behavior of the program processing closer to the actual situation. And lastly, in hybrid analysis, there is an integration of static and dynamic analysis, which makes the detection of Android malware more effective and accurate [10,19].

### Problem Definition and Contributions

Malware is not the only thing that hackers develop. They also try to uncover flaws in the current software and engage in malicious behavior. As a result, it is critical to look for flaws in the Android source code. A coding vulnerability in a program can occur as a result of a mistake made during the design, implementation, or installation phases, and it can be exploited to compromise security. There are two methods for predicting code vulnerabilities. The first approach uses a similar strategy to reverse engineer the APK files (known as PRISMA). The second way involves predicting security problems during the design and development of the programs through the use of Android permissions. Machine-learning classification is highly recommended for the data coming from devices to predict the type of data (malware or benign) and give recommendations about the malware devices. If the data are valid, there is normal traffic, and if the data are invalid, there is malware traffic.

To predict and recommend the Android malware, we have employed static and dynamic analysis using machine-learning models, which shows that our model achieves a high accuracy prediction rate compared to the other models by integrating many characteristics to assist prediction. Taking into account the huge number of malware applications, we require a recommender system that integrates effectively to recognize these applications. Google has identified 10 permissions out of 240 permissions as insecure. Therefore, in this paper, we will extract the important permissions from the applications and utilize the extracted data to effectively detect the malware using a machine-learning model. The main aim of achieving this is to predict the malware with high accuracy, precision, recall, and f1-score. The proposed method examines the permissions and later recognizes the ones that differentiate between benign and malware applications. After extracting the permissions, a machine-learning algorithm will be employed to classify various types of malware and benign applications.

The key contributions are summarized as:

1. To predict the Android malware, our proposed approach has employed static and dynamic analysis using machine-learning models, which shows that the proposed KNN model achieves a 93% accuracy prediction rate compared to the other models by integrating many characteristics to assist the prediction. Hence, a recommendation is provided to the cloud server to block malicious devices.
2. The recommender system is required, which integrates effectively to recognize these applications. Google has found 10 permissions out of 240 permissions as insecure.
3. The proposed algorithm will extract the important permissions from the applications and utilize the extracted data to effectively predict the malware using a machine-learning model.
4. The main aim of achieving this is to predict and recommend the malware with high accuracy, precision, recall, and f1 score. The proposed KNN model examines the permissions and later recognizes the ones that differentiate between benign and malware applications.
5. After extracting the permissions, a machine-learning algorithm will be employed to classify various types of malware and benign applications.

The suggested way represents Android applications using a static and dynamic-based methodology. As a result, illegal apps can be recognized based on their connections to other apps, permissions, and intentions. Android applications are categorized as malicious or benign using the K-nearest neighbor (KNN) method. A machine-learning method called KNN classifies a new app based on the labels of its K closest neighbors after identifying the K apps that are most similar to a given app. A dataset of 1000 Android apps, including 500 malicious and 500 benign apps, was used to assess the suggested strategy. The approach had a 93% success rate in identifying malware. The creation of a feature vector for each app is the initial stage in the KNN algorithm. The features that make up an app’s feature vector can be countless, but some of the more popular ones are the app’s permissions, intents, and API calls. The KNN approach can be used to identify the K most related applications to a given app after the feature vectors for every app in the dataset have been constructed. By calculating the distance between the supplied app and every other app in the dataset, the K most comparable apps are discovered. Different metrics can be used to determine the distance between two apps; however, the Euclidean distance metric is frequently used. Calculating the Euclidean distance between two applications is depicted in Equation (1)

(1) $$distance=sqrt(sum\left({\left(app1-app2\right)}^{2}\right))$$

where the two apps whose distance is being computed are app1 and app2. The labels of the K’s closest neighbors are utilized to categorize the provided app after the K’s most comparable apps have been identified. The provided program is labeled as malware if the majority of its K nearest neighbors are malware. The supplied app is categorized as benign in all other respects.

The steps listed below can be utilized to employ the KNN algorithm to find Android malware in the Internet of Things:

- Assemble a database of Android applications, both malicious and safe.
- For each application in the dataset, create feature vectors.
- Find the K applications that are most similar to each app in the dataset using the KNN algorithm.
- An app is categorized as malware if the majority of its K nearest neighbors are also malicious. The app is categorized as benign on the whole.

The steps listed below can be used to identify Android malware using the KNN algorithm in real time:

1. Check the network for current Android applications.
2. For each new application, create feature vectors.
3. Find the K applications that are most comparable to each new app using the KNN algorithm.
4. A new app is prohibited if the majority of its K nearest neighbors contain malware. Otherwise, the app can be used.

A potential new method for identifying Android malware in the Internet of Things is the KNN algorithm. A quick and effective approach for detecting Android malware in real time is the KNN algorithm.

The association of the work is as follows: Section 2 represents the literature review and analysis, Section 3 shows the framework of proposed Android malware prediction and recommendation for IoT devices, which is followed by Section 4, which includes performance analysis and evaluation metrics, which is followed by the evaluation of the proposed KNN model, and lastly, Section 5 concludes the paper.

## 2. Literature Review and Analysis

In this section, we are focusing on the recent publications which attempted to propose new machine-learning techniques with the leverage of the Android malware detection paradigm. The recent literature on Android malware for IoT devices focuses on benign and malware applications.

The authors of [20] present a deep learning architecture based on the spatial attention and convolutional neural networks (SACNN) for the image-based categorization of 25 well-known malware families with or without category balancing. Precision, recall, specificity, precision, and f1 score were used to assess performance on the Malimg benchmark dataset, and the suggested framework with class balance scored 97.42 percent, 97.95 percent, 97.33 percent, 97.11 percent, and 97.32 percent, respectively.

In [18] This research employs a visualization-based approach in which malware binaries are represented as two-dimensional images and categorized using a deep learning model. We offer a deep learning-based malware detection method that is effective. By managing unbalanced data difficulties, the system uses a re-weighted class-balanced loss function in the final classification layer of the DenseNet model to achieve considerable improvements in performance in classifying malware.

Deduced from nine commercial IoT devices attacked by two botnets: Gafgyt and Mirai, the authors in [21] present a Local–Global Best Bat Algorithm for Neural Networks (LGBA-NN) to choose both feature subsets and hyperparameters enabling the rapid identification of botnet attacks. To modify the bat’s speed in the swarm, the suggested Bat Algorithm (BA) used the local–global best-based inertia weight. To deal with BA swarm diversity, we recommended using a Gaussian distribution for population initialization.

The authors in [13] have found that there are 123,453 benign applications and 5560 malware applications which show 215 API features consisting of eight feature sets and 179 malware families. The objective of using these many features is that Android malware monitoring applications dynamically imply detection patterns and allow malware to be identified directly on the smartphone. The experimental results reflect that the detection rate is 94% of malware families with few false alarms.

There are 31,125 and 11,505 benign and malware applications, respectively, in [22], out of which 420 features are extracted features that deploy a dynamical analytic approach to analyze the effectiveness of DL-Droid through many experimental results. According to our research, DL-Droid can obtain detection rates of up to 97.8% (with dynamic features only) and 99.6% (with dynamic + static features), respectively, outperforming typical machine-learning techniques.

In [23], out of 38,000 and 33,000 benign and malware applications, 32 malware families and 32 feature samples are extracted. The main objective is the autonomous malware detection and attribution system for Android, which uses deep-learning algorithms to classify patterns. For all the tested datasets and conditions, MalDozer can efficiently detect malware and identify them to their legitimate families with an f1 score of 96 percent and a false positive rate of 0.06 percent.

In [24], the 490 and 997 benign and malware applications have nine malware families, out of which eight feature sets are extracted. The main advantage is to address the resource efficiency issue with conventional anti-malware systems, allowing for increased detection performance while maintaining resource consumption.

In [17], 9133 benign and 56 malware families with metadata and apk files are included, from which Plankton is one of the malware families. In this, there is the prediction of Android malware and classifications, apps for mining are used for vulnerabilities, and applications are used for store mining. In [25], 500 and 3723 benign and malware applications are included, out of which 445 feature samples are extracted. The main objective is a client/server module; in order to avoid additional malicious action, users will be automatically notified of the intrusion. MONET can identify malware versions with a 99 percent accuracy rate, according to our tests. Moreover, this can protect against ten different obfuscation and transformation strategies while incurring just roughly 7% computational overhead and 3% energy overhead.

The proposed work overcomes the limitations of the existing literature. To predict the Android malware, our proposed approach has employed static and dynamic analysis using machine-learning models which shows that the proposed KNN model achieves a 93% accuracy prediction rate as compared to the other models by integrating many characteristics to assist the prediction. Hence, a recommendation is provided to the cloud server to block malicious devices. A recommender system is required, which integrates effectively to recognize these applications. Google has found 10 permissions out of 240 permissions as insecure. The proposed algorithm will extract the important permissions from the applications and utilize the extracted data to effectively predict the malware using a machine-learning model. The main aim of achieving this is to predict and recommend the malware with high accuracy, precision, recall, and f1 score. The proposed KNN model examines the permissions and later recognizes the ones that differentiate between benign and malware applications. After extracting the permissions, a machine-learning algorithm will be employed to classify various types of malware and benign applications.

## 3. Framework of Proposed Android Malware Prediction and Recommendation

**Figure 2:**
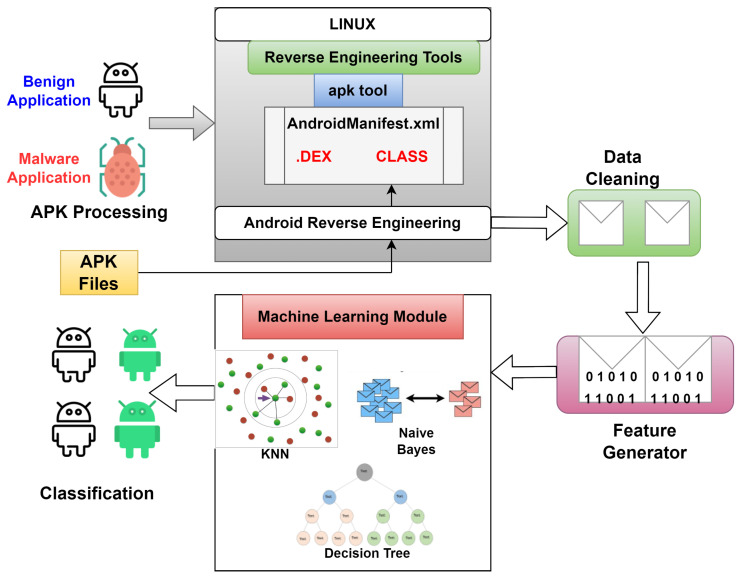
Recommender framework for Android malware detection.

Building upon the drawbacks of the previous work, in this section, as per the static analysis, we have examined some of the features to answer the next hypothesis: there exists a differential of the permissions used between the malware set and benign samples [26]. To fulfill this approach, we have developed a code that comprises the extraction and training of the CSV files, which thereafter has the information regarding the Android application permissions. We can perform the mapping of every APK against a list of permissions. The proposed framework is shown in Figure 2.

### 3.1. Dataset

The dataset employed in this research paper is extracted from Kaggle Android malware (https://www.kaggle.com/datasets/shashwatwork/android-malware-dataset-for-machine-learning, accessed on 19 July 2023). In these malware activities, various APK and permissions are utilized to classify the behavior of ongoing malware applications. Firstly, we have gathered a huge number of Android applications from two different sources. Our script is labeled train.csv, which compares the list of Android permissions and a set of APK’s (malware and benign applications) (https://www.kaggle.com/datasets/xwolf12/datasetandroidpermissions, accessed on 19 July 2023). The second dataset (android_traffic.csv) has been used as a set of Android malware from which each of them was accomplished on the Android virtual devices to generate the network’s traffic. The sample development is about 1.260 malware applications with 49 unique malware families from which 86% are the malware codes that were repackaged versions of legitimate applications, 36.7% could raise the privileges, and 45.3% had the subscription of premium message systems. The four security software were accessed, which could predict 79.6% of the malware applications and 20.2% in the worst case.

### 3.2. Data Preprocessing

Raw feature data can be obtained by extracting features from a dataset; however, these data may not be adequate due to issues such as inaccurate specifications, duplication, missing values, and an imbalanced distribution. Because machine learning on such raw feature data may be unreliable, it is critical to undergo data preprocessing, which is a critical step that underlies machine-learning effectiveness. Data cleaning, data integration, data reduction, and data transformation are examples of data preparation procedures.

### 3.3. Feature Generator

**Figure 3:**
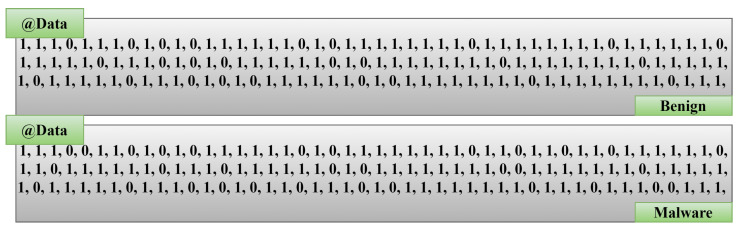
Binary classes for Boolean vectors (malware or benign).

The information was gathered by a procedure that involved creating a binary vector of permissions utilized for each studied program (1 = used, 0 = not used). Furthermore, the malware/benign samples were divided by “Type”: 1 malware and 0 benign. Type is a label, and the total number of 398 applications or AndroidManifest.xml files have been extracted to represent if an application is malware or not. As we can see, the dataset is balanced: 199 applications give rise to value 1, and the rest gives rise to to a value of 0, as depicted in Figure 3.

$X_{a}=1$ means the examiner recognized the permission granted;
$X_{a}=0$ means the permission is not granted.

We have used ApkTool 2.0.3 to acquire the AndroidManifest.xml file of the collected APK. The tools deployed in Python generate the binary vectors that are accessible to the permissions granted for each application. Later, the dataset is generated that comprises the binary values: 0 s or 1 s.

### 3.4. Android Malware Permissions

Android is a popular mobile operating system that is used all over the globe. Android has now become a malware target owing to its technological significance, open-source programming, and the ability to load programs from third parties with no centralized supervision. Even though it has security mechanisms, recent news concerning malicious activities and Android’s shortcomings highlight the significance of continuing to develop approaches and frameworks to enhance its privacy [16].

The Android Application Package (APK) file is a package that comprises each of an Android app’s information. An APK consists of three files and four folders. Our discussion that follows is limited to two key files: classes.dex and AndroidManifest.xml. The Dalvik bytecode, which executes the app’s capabilities, is stored in classes.dex. AndroidManifest.xml is a manifest file containing information regarding the name of the package, version number, points of entry, permissions, and so on. To prevent applications from obtaining system resources, Android uses a permission-based security feature. If an application tries to access hardware and/or software resources, it must use the AndroidManifest.xml file to obtain the necessary permissions. With API calls, permissions are linked to an application’s behaviors and functionalities. In Android’s security framework, the permission mechanism is crucial. For example, if an Android application requires using the Application programming framework’s security-critical or confidentiality functions, it must have the appropriate permissions.

### 3.5. Feature Selection

**Figure 4:**
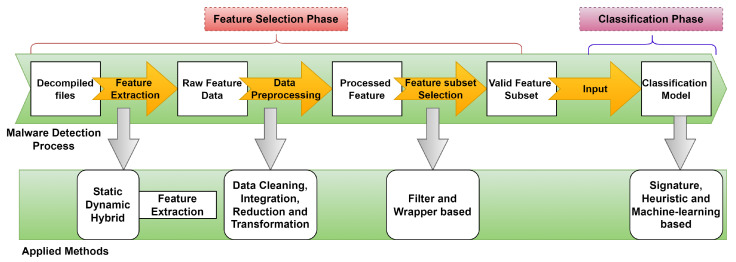
Procedure for feature selection.

When coping with rising data, the selection of features is a crucial data pre-processing phase. This stage aids in the creation of low-complexity models while also ensuring that the data are intelligible. That, in return, enhances the performance of the model by removing noise from the dataset [27]. Moreover, it aids in the prevention of model overfitting and minimizes the time and space complexity of data processing procedures, all of which benefit the model overall. As a result, we divided feature selection into two parts in our research. To begin, we calculated the percentage of each permission across all applications in the dataset, i.e., how many applications specified certain permissions in their manifest files. This gives us an insight into which permissions are employed more commonly than the others as well as which permissions might be working against the model. Secondly, we used backward elimination to exclude features from the classification process that were not statistically important. The steps for selecting the features are shown in Figure 4.

We have induced the top 10 permissions that will be deployed for our benign and malware samples [28]. Some of the most common malware permission are android.permission.

CHANGE_WIFI_STATE,
android.permission.ACCESS_WIFI_STATE,
android.permission.ACCESS_COARSE_LOCATION, etc., and benign permissions are android.permission.WAKE_LOCK,
android.permission.VIBRATE, android.permission.ACCESS_FINE_LOCATION, etc.

**Figure 5:**
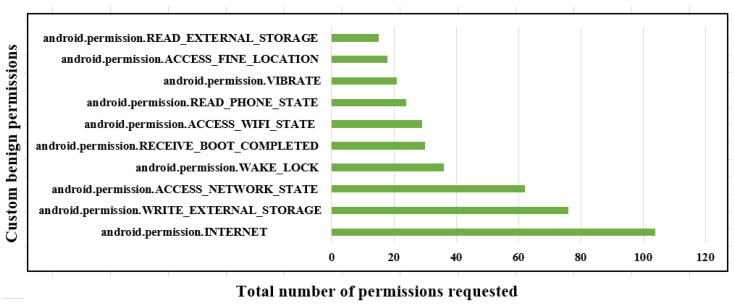
Analysis of Android permissions for benign-based applications.

**Figure 6:**
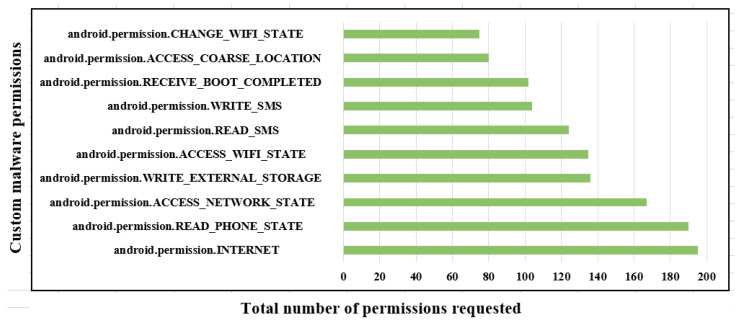
Analysis of Android permissions for malware-based applications.

The results obtained permit us to obtain insights regarding a comparison between the permissions used by the malware and the benign applications, as shown in Table 1 and Figure 5 and Figure 6.

The permissions for datatype int64 in malware follow: the READ_PHONE_STATE has 190 samples, which are more than all the other permissions, and only INTERNET stores 195 samples as per the AndroidManifest.xml file. The value used for datatype int64 is 1, and the axis is 0, which is false and the values are sorted accordingly in the figure shown. The permissions used for benign applications follow: VIBRATE has 21 samples, which is less than the ACCESS_FINE_LOCATION and READ_EXTERNAL_STORAGE as stored in the AndroidManifest.xml file. The value for datatype int64 is 0 and the axis is 1, which is true, and its values are stored in the figures.

### 3.6. Data Modeling

In this data modeling stage, to develop a model for classifying the malware of Android into families, we utilize various classifiers of machine learning. The use of naïve Bayes (NB), random forest (RF), support vector machine (SVM), and the proposed k-nearest neighbors (KNN) model for detecting the Android malware are used. These classification-based algorithms are based on hyperparameters that include accuracy, precision, recall, and f1 score. The KNN classification model challenges are solved using this algorithm. This model applies to the gathered repository of data for creating the model for the detection of malware by classifying APK and permissions into benign and malware classes.

**Figure 7:**
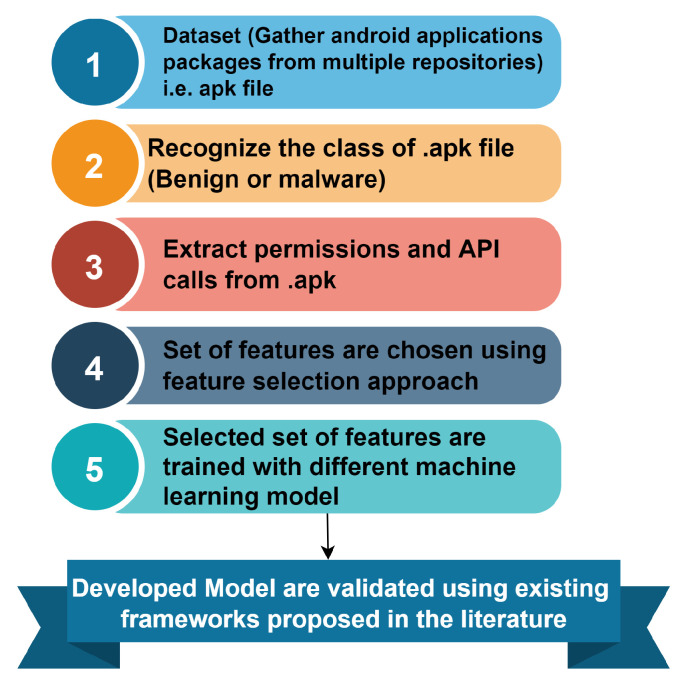
Flowchart of proposed framework.

To classify the data, the K-NN algorithm generates an imaginary boundary. When additional data points are received, the algorithm will attempt to anticipate them as closely as possible to the boundary line. As a consequence, a higher k value indicates smoother separation curves, leading to simpler models. Lower k values, on the other hand, tended to overfit the data, generating complicated models. We integrate the previous data (or train the model) and predict what will happen to employ the k-nearest neighbor approach. The flowchart for the proposed framework is shown in Figure 7, which depicts the dataset that gathers Android applications from various repositories. After gathering the data, the class of .apk file is recognized identifying the benign and malware applications which later extract the permissions and API calls from .apk. The various sets of features are chosen deploying the approach known as feature selection. Lastly, the selected set of features is trained by applying different machine-learning models, and the developed model is validated using the framework proposed in the literature.

The algorithms calculate the distances between a given data point in the set or any K numbers of data points in the dataset that are adjacent to the start position; then, they vote for the classification with the highest frequency. Typically, Euclidean distance (ED) is used as a measuring distance, as mentioned in Equation (2). As a consequence, the outcome is simply labeled data in space. This technique is well known for applications in genomics, prediction, and other fields.

(2) $$ED\;of\;X\;and\;Y=\sqrt{\left(X_{2}-X_{1}\right)+\left(Y_{2}-Y_{1}\right)}$$

For determining the best value of “K”, there is a requirement to try a few values to find the best of them. The value of K is 5, since the value of k = 1 or 2 is the minimum value that can be very noisy, which leads to the effects of outliers in the model.

We are hereby working on the model and want to train it; thus, it is obvious to have a dataset. But after the training, we have to test the model on the same test dataset. The solution is to split the dataset into two sets: a training set and a testing set [29]. We need to make sure that the data should be split randomly: for this, SciKit library provides a tool named Model Selection Library. There is one class in the library which is named “train_test_split”. By using this, we can split the data into training and testing datasets in multiple proportions. There are a few parameters before we create a class:

- test_size: This is the parameter that decides the data size that has to be split as the test dataset. This is assigned as a fraction. For instance, if we pass 0.5 as the value, the dataset will be split, using 50% as the test dataset. If we are specifying this parameter, the next parameter is ignored.
- train_size: There is only a need to specify the parameter if we are not specifying the test_size. It tells what percentage of the dataset you wanted to split as the training set.

We split these two datasets: one is for the feature independent—X, and one is for the variable dependent—Y. We will now split the dataset X into two separate sets— X_train and X_test. In the same way, we will split the dataset Y into two sets—y_train and y_test. In the result analysis, we have split the dataset in 0.9:0.1, 0.8:0.2, and 0.7:0.3, which shows that 90% training and 10% testing give the best results with maximum accuracy, precision, recall, and f1 score. Therefore, we can see how we trained different classifiers to detect malware using its permissions.

## 4. Performance Analysis

### 4.1. Evaluation Metrics

The metrics used for predicting and recommending the Android malware are accuracy, precision, recall and f1 score.

Table 2 shows the run time performance of the proposed model signifying *t*, which means the execution time to complete one detection; *n*, that represents the total number of monitored applications running in the background; *P* shows the run time utilization of a CPU in one round of detection; *Q* shows the run time energy cost in one detection.

### 4.2. Results and Discussion

In this section, the evaluation of the proposed framework for Android malware detection is examined. The performance evaluation of the model is proposed using the accuracy and other metrics for the different values of k [30]. The accuracy, precision, recall, and f1 score for the proposed model on the prediction and recommendation of malware are discussed in Section 4.1. The flowchart of Android malware detection is described as the dataset, gathering the Android applications packages from the numerous repositories i.e., APK files, and then recognizing the different classes of .apk files to verify whether the class is benign and malware, later extracting the Android permissions and API calls from .apk. The set of features is selected by using the feature selection approach [31]. The selected set of features is trained with different machine-learning models, and finally, we developed a KNN model which validates by using the existing frameworks.

We evaluate the outcomes of our suggested model with three alternative methodologies to see if our proposed framework is capable of achieving a higher detection accuracy [4]. We utilized Python as the programming language as well as Scikit-Learn 0.17’s machine-learning features. Naive Bayes, K-nearest neighbor, and decision trees have been the classification algorithms chosen for the study.

### 4.3. Evaluation of Existing Literature with Proposed Model

#### 4.3.1. Evaluation of Proposed KNN Model

**Figure 8:**
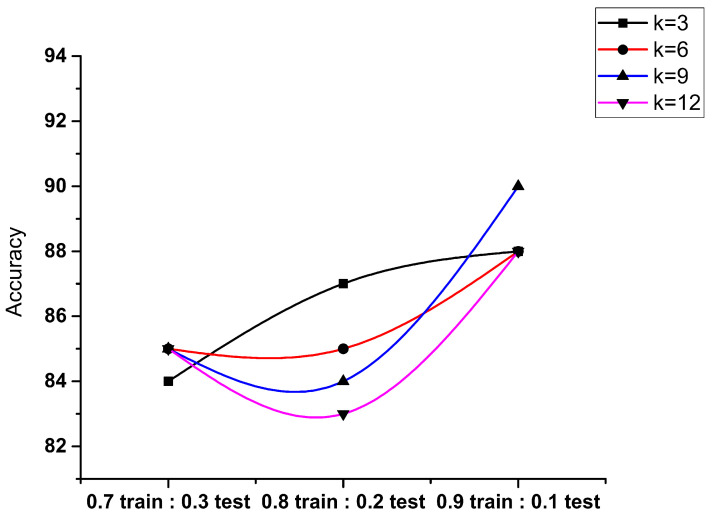
Performance evaluation of KNN accuracy.

We performed four tests with numerous configurations. In Figure 8, we have taken four values of K, i.e., 3, 6, 9, and 12. Three configurations presented samples of 0.7, 0.8, and 0.9 as training data and 0.3, 0.2, and 0.1 as testing data, which had been taken into account for the evaluation of metrics. The accuracy for detecting the Android applications with k = 9 depicts the maximum accuracy having the ratio of 0.9 and 0.1 that shows 91.5%. This means that with k = 9, the detection of Android applications is more accurate compared to the 0.7:0.3 and 0.8:0.2 results.

**Figure 9:**
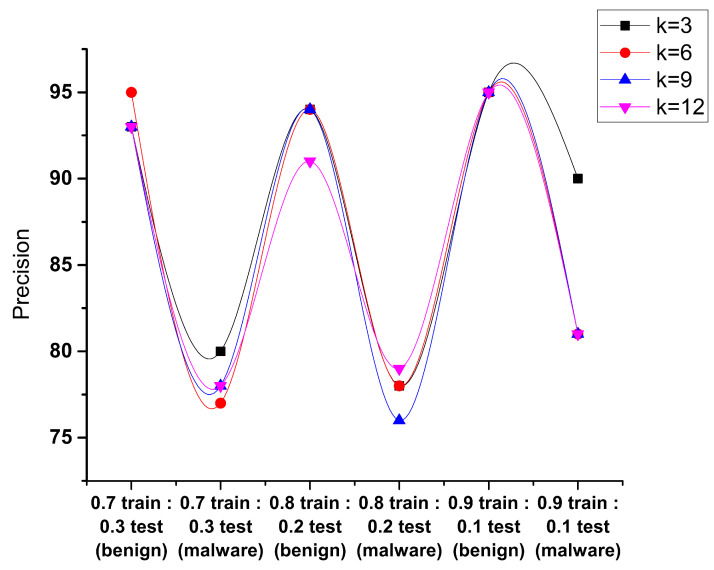
Performance evaluation of KNN precision.

The precision is the total positive predictions for the benign and malware classes. When the samples in Figure 9 were taken as 0.7 and 0.3, the maximum precision came out for the value of k = 3, k = 12 gives the minimum precision for the benign class, and k = 12 gives the minimum precision for the malware class as compared to other values. The samples 0.8 and 0.2 for a benign class give the maximum and minimum precision at the values k = 3 and k = 9, respectively, and for the malware class, the maximum and minimum precision are at the values k = 3 and k = 12, respectively. The results show that when the sample is taken as 0.9 and 0.1, then the precision is achieved with good results.

**Figure 10:**
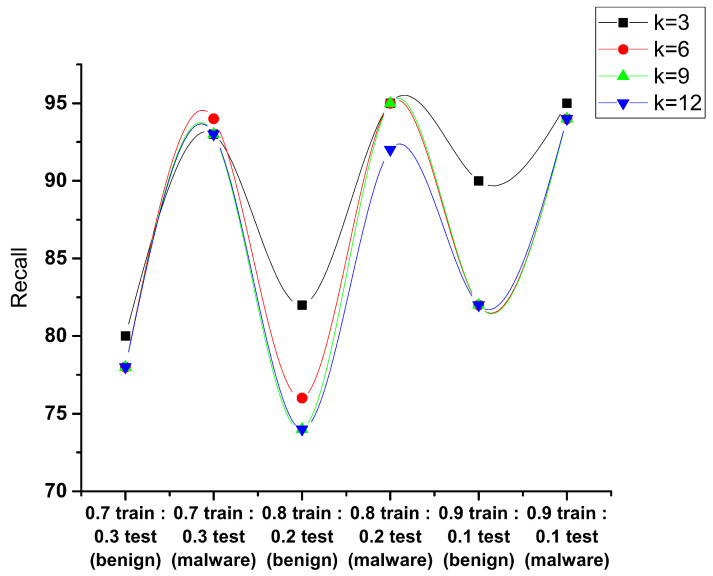
Performance evaluation of KNN recall.

The recall is the total number of predictions carried out from all the positive samples. In Figure 10, to detect the malware as per the samples used for training and testing, it has been observed that 0.9:0.1 achieves the maximum recall of 95% having the value k = 3, which shows the frequency of samples that are correctly detected as malware within all the malware samples that are to be tested.

**Figure 11:**
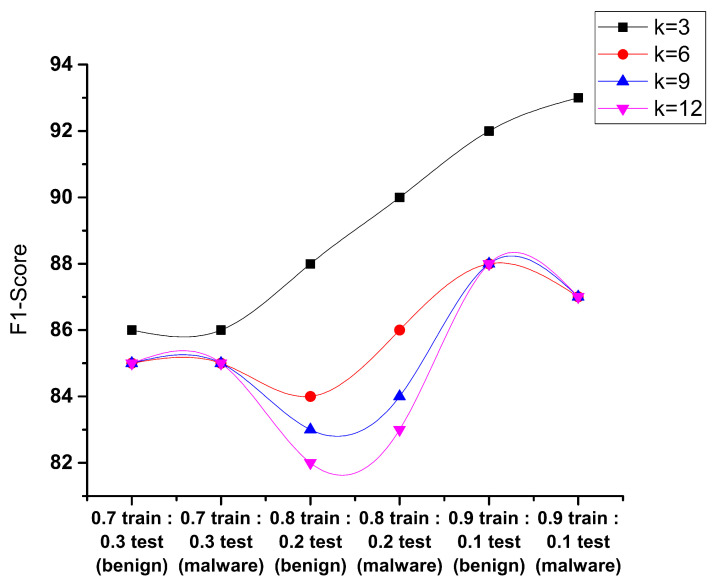
Performance evaluation of KNN f1 score.

The f1 score in Figure 11 is defined as the Android detection malware that is deployed to identify the attack made when the permissions were extracted, and this shows that the 0.9:0.1 sample test gives the best results for benign and malware classes. The f1 score to be achieved with k = 3 gives the maximum f1 score, i.e., 93% compared to the other given samples.

#### 4.3.2. Evaluation of Existing Work with Proposed Model

To provide the significance of the proposed model, its performance is being compared with state-of-the-art detection systems. In this regard, similar approaches are investigated that have been proposed previously. Various kinds of Android malware detection methods were studied. The evaluation has been performed with respect to the metrics used for the comparison. In [32,33], the authors have evaluated the accuracy of KNN with 92% and 91.5%, the precision of KNN with 88% and 87%, the recall of KNN with 89% and 93%, and the f1 score of KNN with 91% and 90% compared to the proposed model. The proposed model shows the handling of multi-features, which further paves the way for its applications for the detection of Android malware detection in IoT devices. Table 3 shows the performance of state-of-the-art approaches with the proposed model.

**Figure 12:**
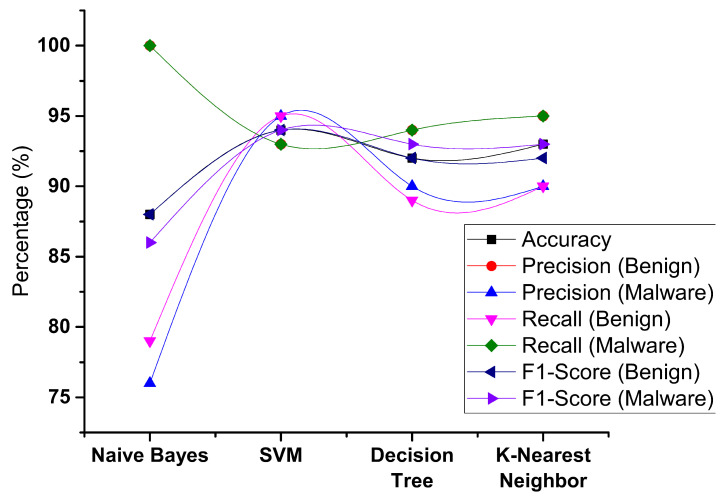
Comparative analysis of various models.

We evaluated some model algorithms in Figure 12 with various setups while using the framework presented in Section 3. The following sections go over the findings of each test. (i) Comparison of results with existing proposed frameworks: To see if our suggested model is capable of detecting malware in the same way as a previously built framework, we calculate two performance parameters for the proposed new model and related models, such as accuracy, recall, precision and f1 score (Table 4). (ii) Detection of known and unknown android malware: Furthermore, we test known and unknown malware categories with our proposed model and estimate the accuracy to detect the malware to see how effective it is in detecting known and new malware categories.

## 5. Conclusions and Future Scope

This paper focuses on developing a malware detection framework that employs a small selection of features to assist us in recognizing whether an Android application is malware or benign. There are a total of 10 different kinds of Android application-based permissions that were used to help with the execution procedure. We provided a methodology for analyzing dangerous applications in Android for evaluation. Four machine-learning methods are used in our approach, and the evaluation is completed based on our dataset results of this experiment employing naive Bayes, K-nearest neighbor, SVM, and decision tree as better classification algorithms. The experimental results shows that the proposed KNN achieves 93%, 95%, 90%, and 92% accuracy, precision, recall and f1 measures, respectively. Our findings were based on applications utilized in prior studies; however, as a novelty, the system provided employs static data to evaluate malware Android applications using machine-learning approaches. It is possible to increase the real-world performance in AI-based applications by integrating the framework with cutting-edge technology to facilitate various computing strategies.

## Figures and Tables

**Table 1:** Android malware permissions.

Malware	Benign
android.permission.INTERNET	android.permission.INTERNET
android.permission.READ_PHONE_STATE	android.permission.WRITE_EXTERNAL_STORAGE
android.permission.ACCESS_NETWORK_STATE	android.permission.ACCESS_NETWORK_STATE
android.permission.WRITE_EXTERNAL_STORAGE	android.permission.WAKE_LOCK
android.permission.ACCESS_WIFI_STATE	android.permission.RECEIVE_BOOT_COMPLETED
android.permission.READ_SMS	android.permission.ACCESS_WIFI_STATE
android.permission.WRITE_SMS	android.permission.READ_PHONE_STATE
android.permission.RECEIVE_BOOT_COMPLETED	android.permission.VIBRATE
android.permission.ACCESS_COARSE_LOCATION	android.permission.ACCESS_FINE_LOCATION
android.permission.CHANGE_WIFI_STATE	android.permission.READ_EXTERNAL_STORAGE

**Table 2:** Run time performance of our proposed model.

CPU Utilization	Memory Utilization	Energy Utilization	Execution Time
$P_{20}\%$	140 ∗ n	$Q_{L}\;*\;t\;*\;n$	infinite

**Table 3:** Comparative analysis of state-of-the-art approaches with proposed model.

Performance Metrics	[32]	[33]	Proposed Model
Accuracy	92%	91.5%	93%
Precision	88%	87%	95%
Recall	89%	93%	95%
F1 Score	91%	90%	92%

**Table 4:** Performance metrics.

Algorithm	Accuracy (%)	Precision (%)	Recall (%)	F1 Measure (%)
Naive Bayes	88	100, 76	79, 100	88, 86
Decision Tree	92	94, 90	89, 94	92, 93
Support Vector Machine	94	93, 95	95, 93	94, 94
K-Nearest Neighbor	93	95, 90	90, 95	92, 93

## Data Availability

Not applicable.
